# Autistic Traits and Attention-Deficit Hyperactivity Disorder Symptoms Associated With Greater Pain Interference and Depression, and Reduced Health-Related Quality of Life in Children With Chronic Pain

**DOI:** 10.3389/fnins.2021.716887

**Published:** 2021-11-01

**Authors:** Camilla Wiwe Lipsker, Tatja Hirvikoski, Leonie J. T. Balter, Sven Bölte, Mats Lekander, Linda Holmström, Rikard K. Wicksell

**Affiliations:** ^1^Department of Clinical Neuroscience, Karolinska Institutet, Stockholm, Sweden; ^2^Center of Neurodevelopmental Disorders (KIND), Centre for Psychiatry Research, Department of Women’s and Children’s Health, Karolinska Institutet, Stockholm Health Care Services, Stockholm, Sweden; ^3^Child and Adolescent Psychiatry, Stockholm Health Care Services, Stockholm, Sweden; ^4^Habilitation and Health, Stockholm, Sweden; ^5^Department of Psychology, Stress Research Institute, Stockholm University, Stockholm, Sweden; ^6^Curtin Autism Research Group, Curtin School of Allied Health, Curtin University, Perth, WA, Australia; ^7^Department of Medical Psychology, Women’s Health and Allied Health Professionals Theme, Karolinska University Hospital, Stockholm, Sweden; ^8^Department of Women’s and Children’s Health, Karolinska Institutet, Stockholm, Sweden; ^9^Pain Clinic, Capio St. Göran’s Hospital, Stockholm, Sweden

**Keywords:** autism spectrum disorder, attention-deficit hyperactivity disorder, chronic pediatric pain, functioning, depression, health-related quality of life, resilience, risk

## Abstract

Previous research indicates elevated levels of clinically significant traits and symptoms of autism spectrum disorder and attention-deficit hyperactivity disorder (ADHD) in children with chronic pain, but associations with functioning and depression are yet unclear. The current study examined the relationships of autistic traits and ADHD symptoms with pain interference, depression, and health-related quality of life, as well as the mediating roles of insomnia and psychological inflexibility, in children with chronic pain (*n* = 146, 8–17 years, 102 girls) presenting at a tertiary pain clinic. Children completed measures of pain intensity, depression, pain interference, health-related quality of life, insomnia, and psychological inflexibility. Parents (*n* = 146, 111 mothers) completed measures to assess autistic traits and ADHD symptoms in their children. Children with clinically significant autistic traits and ADHD symptoms presented with significantly higher levels of depressive symptoms and pain interference, and significantly lower health-related quality of life, than did the other children. Autistic traits and ADHD symptoms contributed significantly to the prediction of pain interference and depressive symptoms, as well as health-related quality of life. Psychological inflexibility mediated the relationships between ADHD symptoms and autistic traits on the one hand and depression, pain interference, and health-related quality of life on the other, while insomnia mediated the relationships between ADHD symptoms and depression, pain interference, and health-related quality of life. All analyses were adjusted for demographics and pain intensity. Results suggest the utility of screening for neurodevelopmental disorders in children with chronic pain. Furthermore, the findings may indicate insomnia and skills related to psychological flexibility as potential treatment targets in interventions aiming at improving functioning and health-related quality of life in children with chronic pain and co-occurring symptoms of neurodevelopmental disorders.

## Introduction

Pediatric chronic pain conditions are prevalent and associated with impaired functioning across physical, emotional, and social domains (Konijnenberg et al., 2005; Petersen et al., 2009b; Hoftun et al., 2011). Such impaired functioning contributes to school absence, disengagement in social activities, and restrictions in physical activities (Sinclair et al., 2016; Groenewald et al., 2020). Children with chronic pain also frequently report significantly lower health-related quality of life (HRQoL) compared to both healthy individuals and children with other chronic conditions (Gold et al., 2009a).

The widely recognized biopsychosocial model of chronic pain (Edwards et al., 2016) emphasizes how biological, psychological, and social factors interplay, vary significantly between individuals, and may influence the development and maintenance of chronic debilitating pain in different ways (Liossi and Howard, 2016). This pattern suggests that some factors may constitute a risk to chronic pain development and functional disability in youth, while others may be protective and promote resilience (Gmuca et al., 2019). For example, sleep disturbances are common and have been associated with an increased risk for both depression and overall functional disability in pediatric chronic pain (Valrie et al., 2013). Importantly, mental health difficulties, such as depression and anxiety (Ryee, 2011), are also highly prevalent in children with chronic pain (Konijnenberg et al., 2006; Tegethoff et al., 2015) and have consistently been related to worse functioning (Hoftun et al., 2011; Caes et al., 2015; Khan et al., 2015; Sinclair et al., 2016). In particular, children with both chronic pain and high levels of depression have been found to be more functionally impaired by their pain symptoms than other children with chronic pain (Kashikar-Zuck et al., 2001). Subjective experience of stress, passive coping strategies, and peer, parent or family negative relationships have also been identified as risk factors associated with chronic pain in youth (McKillop and Banez, 2016). In terms of demographics, relatively older age has been linked to an increased risk for chronic pain development in children and adolescents (Wager et al., 2020), more pain-related impairments among children with severe chronic pain (Zernikow et al., 2012), and lower HRQoL in children with migraine (Powers et al., 2004; Gold et al., 2009b). Chronic pain has also been found to be more common in girls in both community and clinical samples (Konijnenberg et al., 2005; Huguet and Miro, 2008; King et al., 2011; Holmstrom et al., 2015). As these data show, there is a large body of research of specific risk factors that may contribute to pediatric chronic pain and pain-related impairments. The study of factors that can prevent development of severe functional impairments in pediatric chronic pain, and thus constitute resilience factors, is still, however, a neglected research area (Gmuca et al., 2019; Lee et al., 2020).

Resilience is not the absence of risk, but rather the ability to sustain adaptive functioning *despite* significant risk factors being present (Sturgeon and Zautra, 2010). To date, no specific resilience measures have been developed for pediatric chronic pain populations (Gmuca et al., 2019), but factors such as high self-esteem, supportive peer relations, high family cohesion, social competence, and psychological flexibility have been associated with higher levels of functioning among children and adolescents with chronic pain (Wicksell et al., 2009, 2010a; Skrove et al., 2015; Sinclair et al., 2016). Psychological flexibility is defined as the ability to act in accordance with long-term goals in the presence of pain and distress (Hayes et al., 2006; McCracken and Morley, 2014), and thus aims at adaptive functioning despite the presence of pain. Psychological flexibility has accounted for greater functional improvements in both children and adults with chronic pain, as indicated by mediation analyses of exposure- and acceptance-based interventions (Wicksell et al., 2010b, 2011; Feinstein et al., 2011; Feliu-Soler et al., 2018). Taken together, this implies that psychological flexibility may constitute a resilience factor in pediatric chronic pain that may be effectively targeted in psychological treatment. Furthermore, in the healthy population, sleep disturbance and resilience in adolescents have been shown to influence each other over time, where higher levels of sleep disturbance predicted lower resilience to stressful events and vice versa (Wang et al., 2020). Given the association between sleep disturbances and disability in pediatric chronic pain (Pavlova et al., 2017), it is therefore possible that uninterrupted sleep constitutes another resilience factor in pediatric chronic pain, as shown in other pediatric populations (Wong et al., 2018).

Taken together, these data point to the relevance of the continued investigation of potential risk- and resilience factors that may contribute to the understanding of the pediatric chronic pain experience. Greater knowledge and understanding of the relationship between risk- and resilience factors and functioning in pediatric chronic pain will advance the field and provide opportunities for clinical developments, such as identification of potentially important treatment targets (Wojtowicz and Banez, 2015; Liossi and Howard, 2016).

In a recent study including a clinical sample of children and adolescents with debilitating chronic pain, we found elevated levels of clinically significant traits and symptoms of the neurodevelopmental disorders autism spectrum disorder (ASD) and attention-deficit hyperactivity disorder (ADHD) compared to healthy populations. However, only a small portion of children had received a previous diagnosis of either condition (Wiwe Lipsker et al., 2018a). ADHD is defined by age-inappropriate levels of inattention, hyperactivity, and impulsivity, and ASD is characterized by restricted and repetitive behaviors and interests along with social communication and interaction deficits, with a high level of variability in terms of e.g., speech and cognitive abilities (American Psychiatric Association [APA], 2013). Although each disorder is thus characterized by distinct behaviorally defined diagnostic criteria, the DSM-5 specifically allows for a combined diagnosis of the two disorders. To date, there is no agreement concerning the underlying pathology of either ASD or ADHD, where research has suggested that the disorders may share a common origin and manifest along a diagnostic continuum (Kern et al., 2015; Kushki et al., 2019), or be separate disorders with distinct etiological differences (Antshel and Russo, 2019). ASD and ADHD frequently overlap and share deficits in social and executive functioning and other clinical features such as sensory over responsivity, anxiety disorders, such as e.g., obsessive-compulsive disorder, and sleep disorders (Kern et al., 2015; Antshel and Russo, 2019). Similar to pediatric chronic pain, insomnia, and comorbid mental health concerns are highly prevalent among children and adolescents with ASD and ADHD, and have been associated with functional impairments and reduced quality of life (Simonoff et al., 2008; Corkum et al., 2014; Cuffe et al., 2015).

Given these difficulties and based on the above cited research, co-occurring clinically significant symptoms of neurodevelopmental disorders may constitute a risk factor for more functional impairments in pediatric chronic pain (Wiwe Lipsker et al., 2018a; Balter et al., 2021). For the purpose of the current study, we therefore hypothesized that pediatric chronic pain patients with clinically significant traits and symptoms of ASD or ADHD would be at higher risk for worse HRQoL and functional outcomes as compared to other children with chronic pain. Furthermore, we hypothesized that insomnia and psychological inflexibility, as indicators of level of resilience or lack thereof, would statistically mediate the relationship of ASD-traits and ADHD symptoms with pain interference, depressive symptoms and HRQoL.

To this end, the aims of the present study were to examine the relationships of autistic traits and ADHD symptoms, assessed by parents, with pain interference, depressive symptoms, and HRQoL in pediatric chronic pain patients; and to assess the mediating role of insomnia and psychological inflexibility in these relationships. More specifically, we examined (1) if children with scores above cutoff for clinically significant autistic traits or clinically significant symptoms of ADHD differed in functioning and HRQoL as compared to children below the clinical cutoff; (2) if autistic traits and ADHD symptoms explain a significant amount of variance in pain interference, depressive symptoms, and HRQoL, when taking into account age, gender, and pain intensity; and (3) the mediating role of insomnia and psychological inflexibility in the relationships between autistic traits or ADHD symptoms and pain interference, depression, and HRQoL.

## Materials and Methods

### Participants and Procedure

The present study utilized a cross-sectional design and included 146 children and adolescents (henceforth “children” unless otherwise specified) (8–17 years, 102 girls) with chronic pain, consecutively referred to a tertiary pain clinic during a period of 3 years, and their parents (one parent per child, 111 mothers). The sample and procedure has been described previously (Wiwe Lipsker et al., 2018a). In brief, data was collected as a precursor to the initial clinical assessment at the clinic by pen and paper questionnaires. Participants were excluded from the study if they were non-Swedish speakers and unable to complete the questionnaires by themselves. All children and parents provided written and informed consent, and the study was approved by the regional Ethical Review Board in Stockholm.

### Measures

#### Control Variables: Demographics and Pain Intensity (Lübeck Pain Questionnaire) – Child Report

The influence of child demographic variables and pain intensity were adjusted for in the analyses of the relationships between autistic traits and ADHD symptoms and functioning, depression, and HRQoL. Demographic variables were child gender and age (years, months). Pain intensity was assessed using the Lübeck Pain Questionnaire (LPQ) (Roth-Isigkeit et al., 2004, 2005), a structured self-report questionnaire that e.g., evaluates pain intensity, pain duration, pain frequency, pain site/s, perceived trigger/s of pain, and perceived reason/s for first pain onset. For the purpose of the current study, only responses regarding the intensity of pain were analyzed using a visual analog scale (VAS) ranging from “hardly noticeable pain” (0 mm) to “strongest imaginable pain” (100 mm), with six faces ranging from laughter to crying included following the question: “How intense is your main pain usually?”

#### Predictor Variable: Autistic Traits (Social Responsiveness Scale) – Parent Report

The parent-report form of the Social Responsiveness Scale (SRS), a standardized measure of the specific traits and behaviors associated with ASD in 4–18-year old, was used in a Swedish version with the original American norms (Constantino and Gruber, 2005). The psychometric properties of the SRS are well established and the questionnaire comprises 65 items rated on a 4-point Likert Scale ranging from 0 (never true) to 3 (almost always true) (Constantino and Todd, 2003; Constantino and Gruber, 2005; Bolte et al., 2008; Wigham et al., 2012). Raw total scores and total T-scores (norm population *M* = 50, *SD* = 10) were calculated upon completion of all items. Clinically significant ASD symptoms have been associated with T-scores ≥60, and T-scores ≥75 with an ASD diagnosis in the severe range (Constantino et al., 2003). Established thresholds reliably distinguish children with ASD from both non-affected children (sensitivity 0.75 and specificity 0.96) and those with other child psychiatric conditions (sensitivity 0.70 and specificity 0.90) (Constantino and Gruber, 2005). Cronbach’s alpha in the current sample was α = 0.94.

#### Predictor Variable: Attention-Deficit Hyperactivity Disorder Symptoms (Conners 3 ADHD Index) – Parent Report

The parent report form of the Conners 3 (C3), a widely used instrument for assessing symptoms and difficulties associated with ADHD for diagnostic and research purposes (Sparrow, 2010; Schmidt et al., 2017), was used in a Swedish version with comparisons to Swedish norms (Thorell et al., 2015, 2017). The C3 constitutes the updated third edition of the scale and includes a refined assessment of ADHD and updated norms (Conners, 2008). It contains 110-items (108 numeric), where each item is rated on a 4-point Likert-type scale (from 0 = not true at all to 3 = very much true). The C3 also generates an ADHD index that has been shown to accurately differentiate children with ADHD from those without a clinical diagnosis (pooled sensitivities of 0.75 and pooled specificities of 0.75) (Conners, 2008; Chang et al., 2016). Raw total scores and total T-scores for the ADHD-Index were calculated upon completion of all items. T-scores ≥60 indicate an elevated score and T-scores ≥65 are associated with clinically significant ADHD symptoms (Conners, 2008). Cronbach’s alpha in the current sample was α = 0.94.

#### Dependent Variable: Pain Interference Index – Child Report

To assess pain related functioning, children completed the Pain Interference Index (PII), an instrument used to assess the interfering influence of pain on behaviors pertaining to schoolwork, leisure activities, being with friends, physical activities, mood, and sleep (Holmstrom et al., 2015). PII comprises six items rated on a scale from 0 (not at all) to 6 (completely), and the maximum score is 36, where a higher score indicates a higher level of pain interference. The Swedish version of the PII has shown sensitivity to change (Wicksell et al., 2009) and satisfying reliability and internal consistency (Holmstrom et al., 2015; Martin et al., 2015). Cronbach’s alpha for the PII in the current sample was α = 0.86.

#### Dependent Variable: Depressive Symptoms (Center for Epidemiological Studies – Depression Scale Children) – Child Report

Children completed the Center for Epidemiological Studies – Depression Scale Children (CES-DC), which measures symptoms of depression during the last week. The scale was originally designed for adults (Radloff, 1977), and later adapted for use with children and adolescents (Schoenbach et al., 1982). CES-DC is also validated in Swedish (Olsson and von Knotting, 1997). The CES-DC comprises 20 items pertaining to the experience of symptoms of depression ranging from 0 (not at all) to 3 (often) with a maximum score of 60, and higher scores representing more depressive symptoms. A cut-off score of 16 has been suggested for both adult-, child-, and adolescent populations as indicative of significant depressive symptoms (Radloff, 1977; Fendrich et al., 1990). However, this cut-off has been considered too low in child and adolescents populations, that may experience larger mood swings than adults (Olsson and von Knotting, 1997). In order to increase the specificity of the instrument, a higher cut-off of 24 was therefore proposed for children and adolescents in the Swedish validation study (Olsson and von Knotting, 1997). Cronbach’s alpha for the CES-DC in the current sample was α = 0.92.

#### Dependent Variable: Health-Related Quality of Life (Pediatric Quality of Life Inventory 4.0) – Child Report

Children completed the Swedish version of the Pediatric Quality of Life Inventory 4.0 core scales (PedsQL) (Petersen et al., 2009a), which is an instrument for assessing generic health-related quality of life (HRQoL) in children and adolescents (Varni et al., 1999, 2001). The psychometric properties of the PedsQL are well established and it has been used in both healthy children and children with chronic conditions (Varni et al., 2001, 2002, 2003; Gold et al., 2009a), including children and adolescents with chronic pain (Connelly and Rapoff, 2006; Youssef et al., 2006; Gold et al., 2009a). The PedsQL child self-report comes in two developmentally appropriate versions, according to child age: 8–12 and 13–18 years, and due to the age range in the present sample both versions were used. The PedsQL consists of 23 items divided into four subscales: physical health (eight items, Cronbach’s alpha in the current sample was α = 0.85), emotional functioning (five items, α = 0.83), social functioning (five items, α = 0.76), and school functioning (five items, α = 0.78). The total HRQoL scale score is a summary score considering all subscales (23 items, α = 0.92). Each item is rated on a 5-point Likert-type scale assessing how problematic each item has been over the past month (0 = never a problem, 1 = almost never a problem, 2 = sometimes a problem, 3 = often a problem, 4 = almost always a problem). Raw scores are transformed into standard scores from 0 to 100, with a higher score indicating a better HRQoL (Varni et al., 1999). At-risk cutoff scores indicating poor HRQoL for the PedsQL 4.0 core scales have been established by Varni et al. based on a normative sample of healthy children aged 5–18 years: physical health: 72.98; emotional functioning: 59.57; social functioning: 66.61; school functioning: 62.99; and full scale: 69.71 (Varni et al., 2003; Gold et al., 2009b).

#### Mediator: Insomnia Severity Index – Child Report

Children completed a child/adolescent version of the Insomnia Severity Index (ISI) (Bastien et al., 2001), a self-report screening instrument of insomnia. The child version of the instrument has previously been evaluated in a sample of children and adolescents with chronic pain, and was found to be a valid and reliable self-report measure of perceived sleep difficulties (Kanstrup et al., 2014). ISI evaluates sleep during the night, problems with falling asleep, early morning awakenings, satisfaction with the current sleep pattern, interference of insomnia with daily functioning, noticeability by others, and worries about sleep problems during the two past weeks. It consists of seven items that are rated on a scale from 0 (no problems/not at all) to 4 (very much), and the maximum score is 28. The cut-off score for clinically significant insomnia in adolescents (12–19 years) has been suggested as 9 (Chung et al., 2011). Cronbach’s alpha for ISI in the current sample was α = 0.88.

#### Mediator: Psychological Inflexibility in Relation to Pain (Psychological Inflexibility in Pain Scale) – Child Report

Psychological Inflexibility in Pain Scale (PIPS) was used as a self-report measure of psychological inflexibility in relation to experienced pain and related distress (Wicksell et al., 2010a). PIPS assesses avoidance strategies and cognitive fusion, comprising 12 items, such as e.g., “I would do almost anything to get rid of my pain”, rated on a scale from 1 (never true) to 7 (always true). The maximum score is 84, with a higher score indicating a higher level of psychological inflexibility. Cronbach’s alpha for PIPS in the current sample was α = 0.89.

### Statistical Analysis

All analyses were conducted using SPSS version 25 (IBM, 2017). Analysis of missing data and variable distributions were performed. The expectation maximization algorithm (EM) (Dempster et al., 1977) was employed for imputing missing data for all variables except autistic traits (SRS) and ADHD symptoms (C3), where missing data was imputed according to instructions in the respective manual (Constantino and Gruber, 2005; Thorell et al., 2015).

The assumption of normality was tested for each variable through visual inspection of histograms, Q-Q plots, and calculations of absolute z-scores for both skewness and kurtosis compared to critical values according to sample size (Kim, 2013). Results showed that the sample regarding pain interference (PII), depression (CES-DC), HRQoL (PedsQL), insomnia (ISI), and psychological inflexibility (PIPS) were approximately normally distributed, while the distributions of ADHD symptoms (C3) and autistic traits (SRS) were not. As the general linear model makes no distributional assumptions about the independent variables (IV) other than independence between the IV and the error (Fox, 2015), independence was checked using the Durbin-Watson statistic and visual inspection of scatter plots of standardized residuals and standardized predicted values. Analyses showed that the assumptions of independence and homoscedasticity, respectively, had been met for all dependent variables (DV) used in the analyses (obtained Durbin-Watson values were, e.g., close to 2 for all DVs).

Descriptive statistics and Pearson’s product moment correlations (*r*) were used to examine data and evaluate the relationships between all variables. A correlation of *r* = ± 0.00 −0.29 was considered weak, *r* = ± 0.30 −0.49, moderate, *r* = ± 0.50 −0.89, strong, and *r* ≥ ± 0.90, very strong (Field, 2013).

Independent-samples *t*-tests were used to compare the children below and above cutoff for clinically significant autistic traits (SRS) or ADHD symptoms (C3) for HRQoL (PedsQL), pain interference (PII), and depression (CES-DC). Total and subscale PedsQL mean scores and CES-DC mean scores were referenced against established at-risk and cutoff mean scores for these instruments. Hedges’ g was used as a measure of effect size due to unequal sample sizes in the groups below and above cutoff for clinically significant autistic traits (SRS) and symptoms of ADHD (C3). Effect sizes were considered small (*g* = 0.3), medium (*g* = 0.5) and large (*g* = 0.8) (Wasserman et al., 1988).

Hierarchical linear regression analyses were performed to investigate the amount of unique variance explained by autistic traits (SRS) and ADHD symptoms (C3) in HRQoL (PedsQL), depression (CES-DC), and pain interference (PII), respectively. Age and gender were included in the hierarchical regression model as step 1, followed by pain intensity (LPQ) (step 2) and symptoms and traits of autism or ADHD (SRS and C3) (step 3). Effect sizes were considered small (*F*^2^ = 0.02), medium (*F*^2^ = 0.15) and large (*F*^2^ = 0.35) (Cohen, 1988). A conventional level for statistical significance was set at *p* < 0.05.

Finally, indirect effects of insomnia (ISI) and psychological inflexibility (PIPS) in the relationships between the predictors, i.e., autistic traits (SRS) or ADHD symptoms (C3), and dependent variables, i.e., depression (CES-DC), pain interference (PII) and HRQoL (PedsQL), were evaluated. A series of analyses were conducted using the PROCESS macro for SPSS. PROCESS is a bootstrapping method in which samples of the original size, drawn from the original data, are generated (Hayes and Rockwood, 2017), and for the purpose of the current study a bootstrap method with *n* = 5,000 bootstrap resamples and 99% bias-corrected and accelerated confidence intervals was used. First, insomnia (ISI) was included as the mediator (M), with symptoms of ADHD (C3) entered as the predictor (X) and depression (CES-DC), pain interference (PII) and HRQoL (PedsQL) as dependent variables (Y) in separate mediation analyses. Subsequently, autistic traits (SRS) was used as the predictor with the same set of dependent variables. In all analyses the influence of age, gender, and pain intensity (LPQ) were adjusted for by entering these variables as covariates. Second, psychological inflexibility (PIPS) was used as mediator, following the same procedure as described above for insomnia (ISI) using the same predictors, dependent variables, and covariates. The total effect (*c*) is comprised of the direct effect (*c*′) and the indirect effect (*ab*). Thus, the indirect effect represents the part of the relation between the predictor and the dependent variable that can be explained by the proposed mediator. The mean value for the *ab* product across the bootstrapped samples provided a point estimate of the indirect effect. Confidence intervals (CI) were derived from the obtained distribution of a and b scores, using a 99% CI level representing a significance level of *p* < 0.01. If lower and upper bounds did not contain zero, the indirect effect was significant at the specified level. Each analysis was based on 5000 bootstrapped samples, as suggested by Preacher and Hayes (2008).

### Power Calculations

Power calculations were performed based on research questions 1 and 2. In a *post hoc* analysis for independent sample *t*-tests (research question 1) with an unequal allocation of participants in the groups below and above cutoff for clinically significant autistic traits (SRS) and symptoms of ADHD (C3) (38 and 108, respectively, based on actual sample sizes), the achieved power was β = 0.19 (power 81%) using α = 0.05, a medium effect size (*g* = 0.5), and two tails (Faul et al., 2007). Using an *a priori* power analysis for linear multiple regression: fixed model, single regression coefficient with five predictors in the model (research question 2), calculations for β = 0.2 (power 80%) and α = 0.05, indicated a minimum of *n* = 55 to detect a medium effect size (*F*^2^ = 0.15) (Faul et al., 2007). Actual power based on the sample size 146 was β = 0.01 (power 99%) using α = 0.05, effect size (*F*^2^ = 0.15), and two tails. In conclusion, the current study was sufficiently powered to detect statistically significant differences.

## Results

### Sample Characteristics, Pain (LPQ), and Clinically Significant Autistic Traits (SRS) and ADHD Symptoms (C3)

Child and parent demographics (for details, please see Table S1 in Wiwe Lipsker et al., 2018a), child pain (LPQ; for details, please see Table S2 in Wiwe Lipsker et al., 2018a), and levels of clinically significant autistic traits (SRS) and symptoms of ADHD (C3), have been reported in detail elsewhere (Wiwe Lipsker et al., 2018a). In brief, mean age was 14.55 years (*SD* = 2.40) for the children. Pain duration of more than 6 months was reported by 93.8% of the children, and the remaining 6.2% reported pain during at least 3 months. The average pain intensity (LPQ) was 59.02 (*SD* = 13.55; max = 100). Furthermore, 69.2% of the children reported being in pain every day and 24% reported pain several times a week. A total of 74% had pain from three or more body sites. Headache was the most prevalent pain type affecting 79.5% of children, followed by back pain in (61%), stomach pain (59.6%), and leg pain, reported by 48.6% of all children (Wiwe Lipsker et al., 2018a).

Regarding traits and symptoms of autism and ADHD, scores consistent with clinically significant autism (SRS) were reported for 20 children (13.7%) and scores consistent with clinically significant ADHD (C3) were reported for 29 children (19.9%). In total, clinically significant traits and symptoms of either or both autism/ADHD (SRS/C3) were reported for 38 children (26%) (Wiwe Lipsker et al., 2018a).

### Missing Data

Of 223 potential participants that were approached, 190 parent-child dyads agreed to participate by completing the informed consent forms. Of these, 16 parent-child dyads did not complete the questionnaires, and an additional 28 dyads returned incomplete (only child or only parent) measures and were subsequently excluded. Finally, data from 146 parent-child dyads were included in the analyses.

Less than 0.5% of values were missing on either SRS and C3, and imputation of missing data was performed according to scoring instructions in the respective manual for each instrument (Constantino and Gruber, 2005; Thorell et al., 2015). Across all child forms, 0.7% of data was missing in PedsQL, 2.4% in PII, 0.5% in CES-DC, 1% in ISI, and 0.6% in PIPS. For all instruments, missing data was found to be missing completely at random (Little’s MCAR = ns) (Little, 1988). Imputations of missing data in PedsQL, PII, CES-DC, ISI, and PIPS were performed using the EM-algorithm. For LPQ, data was missing for 6 participants (4%) and these were not imputed.

### Descriptive Statistics and Bivariate Correlations

Means, standard deviations, and bivariate correlations between control-, predictor-, dependent variables, and mediators are presented in Table 1. In brief, 58% of children reported scores on the PedsQL equal to or lower than the at-risk score for poor HRQoL (69.71) (Varni et al., 2003; Gold et al., 2009b), and 35% received scores of 24 or above on the CES-DC, indicative of clinically significant depressive symptoms (Olsson and von Knotting, 1997). On ISI, 43% received scores at 9 or above indicating clinically significant insomnia (Chung et al., 2011).

**TABLE 1:** Descriptive statistics and correlations between control, predictor, and dependent variables and mediators (*n* = 146^a^).

**Variables**	**Mean (%)**	***SD***	**Gender**	**Age**	**Pain intensity**	**C3-T**	**SRS-T**	**PII**	**CES-DC**	**PedsQL**	**ISI**
1. Gender (F, M)	(70, 30)										
2. Age	14.55	2.40	0.018								
3. Pain intensity	59.02	13.55	0.068	0.047							
4. C3 T-scores	55.42	12.82	0.041	0.096	–0.005						
5. SRS T-scores	48.04	10.40	0.16	0.163*	0.075	0.575**					
6. PII	15.27	9.82	0.143	0.198*	0.267**	0.307**	0.341**				
7. CES-DC	18.75	12.75	0.137	0.206*	0.157	0.358**	0.375**	0.753**			
8. PedsQL (23 items)	62.03	18.64	−0.176*	−0.175*	−0.232**	−0.352**	−0.365**	−0.845**	−0.822**		
9. ISI	8.11	6.4	0.155	0.208*	0.204*	0.224**	0.177*	0.575**	0.603**	−0.665**	
10. PIPS	45.66	15.73	0.018	0.196*	0.304**	0.289**	0.378**	0.720**	0.634**	−0.682**	0.373**

*^*a*^Pain intensity, *n* = 140 measured with the Lübeck Pain Questionnaire (LPQ); C3, Conners 3; SRS, The Social Responsiveness Scale; PII, Pain Interference Index; CES-DC, Center for Epidemiological Studies – Depression Scale Children; PedsQL, Pediatric Quality of Life Inventory; ISI, Insomnia Severity Index; PIPS, Psychological Inflexibility in Pain Scale.*

*Correlation is significant at the 0.05 level (two-tailed).

**Correlation is significant at the 0.01 level (two-tailed).

Both ADHD symptoms (C3) and autistic traits (SRS) showed significant positive correlations with depression (CES-DC) (C3: *r* = 0.36, *n* = 146, *p* < 0.001 SRS: *r* = 0.38, *n* = 146, *p* < 0.001) and pain interference (PII) (C3: *r* = 0.31, *n* = 146, *p* < 0.001; SRS: *r* = 0.34, *n* = 146, *p* < 0.001). Thus, higher levels of autistic traits and ADHD symptoms were, to a moderate degree, associated with higher levels of depressive symptoms and pain interference. Furthermore, results revealed significant negative correlations between ADHD symptoms (C3) and autistic traits (SRS) and the full scale for HRQoL (PedsQL) (C3: *r* = –0.35, *n* = 146, *p* < 0.001; SRS: *r* = –0.36, *n* = 146, *p* < 0.001). Higher levels of autistic traits and ADHD symptoms were associated with lower levels of HRQoL to a moderate degree.

Both ADHD symptoms (C3) and autistic traits (SRS) also showed significant weak positive correlations with insomnia (ISI) and positive, moderate in strength for autistic traits (SRS) and weak for ADHD symptoms (C3), correlations with psychological inflexibility (PIPS). Insomnia (ISI), pain interference (PII), and depressive symptoms (CES-DC) were furthermore strongly related to each other, where more severe insomnia was associated with more depressive symptoms, greater pain interference, and reduced HRQoL and vice versa. Descriptive statistics and results for bivariate correlations are presented in Table 1.

### Comparison of Children Below and Above Cutoff for Clinically Significant Autistic Traits (SRS) and/or ADHD Symptoms (C3) for Pain Interference (PII), Depression (CES-DC), and HRQoL (PedsQL)

Children above cutoff for clinically significant traits and symptoms of either autism (SRS) or ADHD (C3) reported lower HRQoL on the PedsQL full scale (*M* = 50.6, *SD* = 18.65) than did those below cutoff (*M* = 66.05, *SD* = 16.97), *t*(144) = 15.448, *p* < 0.001, *g* = 0.9. Also, scores for the above cutoff-group were significantly lower compared to children below the clinical cutoff score on all four PedsQL subscales, with large effects for emotional-, social-, and school functioning. Children above cutoff for clinically significant autistic/ADHD-traits and symptoms (SRS/C3) also reported higher levels of pain interference (PII) (*M* = 20.45, *SD* = 8.41) than children below cutoff (*M* = 13.45, *SD* = 9.66), *t*(144) = −7.003, *p* < 0.001, *g* = 0.7, and more depressive symptoms (CES-DC) (*M* = 26.76, *SD* = 14.12) than children below cutoff (*M* = 15.94, SD = 10.97), *t*(53.558) = −10.822, *p* < 0.001, *g* = 0.9. These effects were considered large.

Referencing the results for above/below cutoff for clinically significant autistic/ADHD-traits and symptoms (SRS/C3) to at-risk cutoffs for HRQoL (PedsQL) and depression (CES-DC), 79% of children above autism/ADHD (SRS/C3) cutoff reported scores on the PedsQL equal to or lower than the at-risk level for poor HRQoL (children below autism/ADHD cutoff, 50%) (Varni et al., 2003; Gold et al., 2009b), and 58% received scores of 24 and above on the CES-DC, indicative of significant depressive symptoms (children below autism/ADHD cutoff, 28%) (Olsson and von Knotting, 1997). Notably, scores for school functioning and health on the PedsQL were particularly low in both groups, compared to at-risk scores, although much lower in the above autism/ADHD (SRS/C3) cutoff group. Results from *t*-tests are presented in Table 2.

**TABLE 2:** Independent groups *t*-tests between variables related to pain interference (PII), depression (CES-DC), and HRQoL (PedsQL) and cutoff status for clinically significant traits and symptoms of either autism (SRS) or ADHD (C3) (*n* = 146).

	**Below cutoff C3/SRS (*n* = 108)**		**Above cutoff C3/SRS (*n* = 38)**					
**Variables**	**Mean**	***SD***	**Mean**	***SD***	***df***	***t***	**Sig.**	**Effect size (*g*)**
PedsQL full	66.05	16.97	50.6	18.65	144	15.448	0.000	0.9
PedsQL health	57.64	21.48	46.52	22.3	144	11.124	0.008	0.5
PedsQL emotional	70.97	21.93	50.98	25.2	144	19.989	0.000	0.9
PedsQL social	83.14	16.58	67.86	22.89	144	15.282	0.000	0.8
PedsQL school	57.48	22.47	39.49	21.49	144	17.993	0.000	0.8
PII	13.45	9.66	20.45	8.41	144	–7.003	0.000	0.7
CES-DC^a^	15.94	10.97	26.76	14.12	53.558	–10.822	0.000	0.9

*PII, Pain Interference Index; CES-DC, Center for Epidemiological Studies – Depression Scale Children; PedsQL Full, Pediatric Quality of Life Inventory, full scale; PedsQL Health, Pediatric Quality of Life Inventory, physical health scale; PedsQL Emotional, Pediatric Quality of Life Inventory, emotional functioning scale; PedsQL Social, Pediatric Quality of Life Inventory, social functioning scale; PedsQL School, Pediatric Quality of Life Inventory, school functioning scale.*

*^*a*^Reporting results from output ‘equal variances not assumed’, Levene’s test for equal variances = Sig. > 0.05 for all variables except CES-DC = Sig. 0.007; C3, Conners 3; SRS, The Social Responsiveness Scale.*

### Regression Analyses Examining the Relationship Between Autistic Traits (SRS) and ADHD-Symptoms (C3) and Pain Interference (PII), Depression (CES-DC), and HRQoL (PedsQL)

Hierarchical regression analyses showed that ADHD symptoms (C3) and autistic traits (SRS) contributed significantly to the prediction of HRQoL (PedsQL) (*p* < 0.001), depressive symptoms (CES-DC) (*p* < 0.001), and pain interference (PII) (*p* < 0.001). ADHD symptoms (C3) and autistic traits (SRS) accounted for 14.3% of the variation in HRQoL (PedsQL), 15.2% of the variation in depression (CES-DC), and 11.3% of the variation in pain interference (PII) with adjustment for age, gender, and pain intensity (LPQ). Concerning the individual contributions of ADHD symptoms (C3) and autistic traits (SRS), both variables obtained significant beta coefficients for all dependent variables. The results from the hierarchical regression analyses are presented in Table 3.

**TABLE 3:** Hierarchical linear regression analyses examining the association between autistic traits (SRS) and ADHD symptoms (C3) on pain interference (PII), depression (CES-DC), and HRQoL (PedsQL) (*n* = 146^a^), adjusted for gender, age, and pain intensity (LPQ).

							**Unstandardized^b^**		**Standardized coefficients Beta^b^**		
**Dependents**	**Step**	**Predictors**	***R* ^2^**	***R*^2^ Change**	***F* Change (*df*)**	**Sig. *F* Change**	**B**	**SE**	**β**	***t***	**Sig.**
PedsQL	1	Controls	0.085	0.085	6.32 (2,137)	0.002					
		Gender					−6.058	3.049	−0.149	−1.99	0.049
		Age					−1.13	0.584	−0.144	−1.93	0.055
	2	Pain	0.128	0.044	6.84 (1,136)	0.01					
		Pain intensity					−0.278	0.103	−0.201	−2.71	0.008
	3	ASD/ADHD	0.271	0.143	13.13 (2,134)	0.000					
		C3					−0.326	0.131	−0.227	−2.50	0.014
		SRS					−0.364	0.165	−0.205	−2.21	0.029
CES-DC	1	Controls	0.084	0.084	6.27 (2,137)	0.002					
		Gender					3.12	2.107	0.112	1.48	0.141
		Age					0.943	0.404	0.176	2.34	0.021
	2	Pain	0.102	0.018	2.75 (1,136)	0.099					
		Pain intensity					0.118	0.071	0.125	1.66	0.099
	3	ASD/ADHD	0.254	0.152	13.61 (2,134)	0.000					
		C3					0.213	0.09	0.217	2.36	0.02
		SRS					0.277	0.114	0.228	2.44	0.016
PII	1	Controls	0.08	0.08	5.92 (2,137)	0.003					
		Gender					2.426	1.63	0.113	1.49	0.139
		Age					0.692	0.312	0.167	2.22	0.028
	2	Pain	0.14	0.061	9.59 (1,136)	0.002					
		Pain intensity					0.174	0.055	0.238	3.16	0.002
	3	ASD/ADHD	0.253	0.113	10.16 (2,134)	0.000					
		C3					0.142	0.07	0.186	2.02	0.045
		SRS					0.187	0.088	0.199	2.12	0.036

*^*a*^Pain intensity, *n* = 140 measured with the Lübeck Pain Questionnaire (LPQ).*

*^*b*^Results are displayed from the final model.*

*C3, Conners 3; SRS, The Social Responsiveness Scale; PII, Pain Interference Index; CES-DC, Center for Epidemiological Studies – Depression Scale Children; PedsQL, Pediatric Quality of Life Inventory; Sig., significance.*

### Analyses of the Mediating Role of Insomnia (ISI) in the Relationships Between Autistic Traits (SRS) and ADHD Symptoms (C3), Depression (CES-DC), Pain Interference (PII), and HRQoL (PedsQL)

The bootstrap method illustrated significant indirect effects of insomnia (ISI) (M) in the relationships between ADHD symptoms (C3) (X) and the three dependent variables (Y): depression (CES-DC), pain interference (PII), and HRQoL (PedsQL) (*p* ≤ 0.01). Analyses also showed significant direct effects (*c*′). Thus, insomnia (ISI) represents a partial mediator in all three models with ADHD symptoms (C3) as predictor and depression (CES-DC), pain interference (PII), or HRQoL (PedsQL) as dependent variables, adjusted for the influences of gender, age, and pain intensity (LPQ).

In contrast, no significant indirect effects were seen for insomnia (ISI) (M) in any of the relationships between autistic traits (SRS) (X) and depression (CES-DC), pain interference (PII), or HRQoL (PedsQL) (Y). Results are summarized in Table 4.

**TABLE 4:** Total, direct and indirect effects of autistic traits (SRS) and ADHD symptoms (C3) on depression (CES-DC), pain interference (PII), and HRQoL (PedsQL), using insomnia (ISI) as mediator with adjustment for age, gender, and level of pain intensity (LPQ) (*n* = 146).

**Mediator: ISI**						**Indirect effect^a^**		
***X***	***Y***	***a* path coefficient**	***b* path coefficient**	**Total effect (c)**	**Direct effect (c′)**	**Effect (SE)**	**CI (99%)**	
							**LLCI**	**ULCI**
C3	CES-DC	0.102*	1.053**	0.340**	0.233**	0.108** (0.043)	0.005	0.230
	PII	0.102*	0.733**	−0.227**	0.152**	0.075** (0.030)	0.006	0.167
	PedsQL	0.102*	1.718**	−0.494**	−0.318**	–0.176** (0.067)	–0.364	–0.014
SRS	CES-DC	0.070	1.090**	0.432**	0.356**	0.076 (0.052)	–0.057	0.228
	PII	0.070	0.757**	0.290**	0.237**	0.053 (0.036)	–0.048	0.149
	PedsQL	0.070	−1.769**	−0.602**	−0.478**	–0.123 (0.085)	–0.354	0.097

*Number of bootstrap resamples = 5000; Covariates in all analyses: age, gender, pain intensity; LLCI/ULCI, Lower/Upper Level Confidence Interval; C3, Conners 3; SRS, The Social Responsiveness Scale; PII, Pain Interference Index; CES-DC, Center for Epidemiological Studies – Depression Scale Children; PedsQL, Pediatric Quality of Life Inventory.*

^*a*^The indirect effect is statistically significant when the confidence interval (LLCI – ULCI) does not include zero (99% equals *p* < 0.01 level significance). *p < 0.05 and **p < 0.01.

### Analyses of the Mediating Role of Psychological Inflexibility (PIPS) in the Relationships Between Autistic Traits (SRS) and ADHD Symptoms (C3), Depression (CES-DC), Pain Interference (PII), and HRQoL (PedsQL)

The bootstrap method illustrated significant indirect effects (*p* ≤ 0.01) of psychological inflexibility (PIPS) (M) in all relationships between both predictor variables (ADHD symptoms (C3) and autistic traits (SRS), respectively) (X) and the dependent variables [depression (CES-DC), pain interference (PII), and HRQoL (PedsQL)] (Y). In three models, the direct effects (*c*′) were non-significant indicating a strong mediating effect, and in one model the direct effect was significant at *p* ≤ 0.05. Thus, psychological inflexibility (PIPS) mediated the relationships between autistic traits (SRS) as well as ADHD symptoms (C3) and the respective dependent variables depression (CES-DC), pain interference (PII), and HRQoL (PedsQL), when the influences of gender, age, and pain intensity (LPQ) were adjusted for. Results are summarized in Table 5.

**TABLE 5:** Total, direct and indirect effects of autistic traits (SRS) and ADHD symptoms (C3) on depression (CES-DC), pain interference (PII), and HRQoL (PedsQL), using psychological inflexibility (PIPS) as mediator with adjustment for age, gender, and level of pain intensity (LPQ) (*n* = 146).

**Mediator: PIPS**						**Indirect effect^a^**		
***X***	***Y***	***a* path coefficient**	***b* path coefficient**	**Total effect (c)**	**Direct effect (c′)**	**Effect (SE)**	**CI (99%)**	
							**LLCI**	**ULCI**
C3	CES	0.342**	0.454**	0.340**	0.185**	0.155** (0.044)	0.052	0.282
	PII	0.342**	0.414**	0.227**	0.086	0.142** (0.036)	0.050	0.240
	PedsQL	0.342**	−0.722**	−0.494**	−0.247**	–0.247** (–0.069)	–0.439	–0.079
SRS	CES	0.556**	0.455**	0.432**	0.180*	0.253** (0.056)	0.114	0.409
	PII	0.556**	0.422**	0.290**	0.055	0.235** (0.048)	0.109	0.362
	PedsQL	0.556**	0.734**	−0.602**	–0.194	–0.408** (0.087)	–0.661	–0.184

*Number of bootstrap resamples = 5000; Covariates in all analyses: age, gender, pain intensity; LLCI/ULCI, Lower/Upper Level Confidence Interval; C3, Conners 3; SRS, The Social Responsiveness Scale; PII, Pain Interference Index; CES-DC, Center for Epidemiological Studies – Depression Scale Children; PedsQL, Pediatric Quality of Life Inventory.*

^*a*^The indirect effect is statistically significant when the confidence interval (LLCI – ULCI) does not include zero (99% equals *p* < 0.01 level significance). *p < 0.05 and **p < 0.01.

## Discussion

In the present study, we examined the relationships of autistic traits and ADHD symptoms with functioning (pain interference), depression, and HRQoL in a sample of children and adolescents presenting at a tertiary pain clinic for chronic pain.

Autistic traits and ADHD symptoms were found to predict pain interference and depression, as well as reduced HRQoL, adjusted for demographics and pain intensity. Overall, results provide additional support for the previously documented relationships between pediatric chronic pain, impaired functioning, and poor HRQoL (Palermo and Kiska, 2005; Gold et al., 2009a,b; Petersen et al., 2009b; Ingerski et al., 2010; Valrie et al., 2013; Kanstrup et al., 2014), and further emphasize the burden that many of these children endure. However, and in line with our hypothesis, it was also found that children and adolescents with chronic pain and clinically significant traits of autism (T-scores ≥60) or ADHD symptoms (T-scores ≥65) displayed significantly higher levels of depressive symptoms and pain interference, and significantly lower HRQoL, than did the other children in the sample.

Although, the causal direction of these relationships remains unclear, results nevertheless indicate that co-occurring clinically significant traits and symptoms of a neurodevelopmental disorder may constitute a risk factor for more severe functional impairment in pediatric chronic pain. Considering the high sensitivity and specificity of the C3 (Conners, 2008; Chang et al., 2016) and the SRS (Constantino and Gruber, 2005), in relation to measuring traits and symptoms of neurodevelopmental disorders with a likely hereditary pathogenesis (Ghirardi et al., 2018), it is therefore possible that having clinically significant autistic traits and ADHD symptoms not only poses an increased risk of presenting with chronic pain (Wiwe Lipsker et al., 2018a), but also with more functional disability (Wiwe Lipsker et al., 2018b; Balter et al., 2021) than other children with chronic pain. Research also suggests possibly shared neuropathological processes between chronic pain and neurodevelopmental disorders. For instance, hypodopaminergic state (low dopamine function) and sensory abnormalities, both present in chronic pain conditions (Niikura et al., 2010; Taylor et al., 2016; Borsook et al., 2018), have also been documented in ASD and ADHD (Reynolds and Lane, 2009; Mazurek et al., 2012; Gold et al., 2014). In chronic pain, the hypodopaminergic state has e.g., been shown to impair rewarded behavior and has been suggested as a possible factor in the development of depression (Taylor et al., 2016).

We furthermore found insomnia to be a mediator of the relationships between ADHD symptoms and HRQoL, depression, and pain interference, but not of the relationships between these dependent variables and autistic traits. These findings are in line with research suggesting a close connection between ADHD and sleep disturbances that is hypothesized to contribute, and be directly linked, to some of the core ADHD symptoms, possibly due to disruptions of circadian rhythms (Coogan et al., 2016). Sleep problems have also been found to independently predict lower levels of QoL in children with ADHD (Craig et al., 2020), and the specific targeting of sleep within a risk- and resilience framework has been suggested as an important development in future ADHD-management (Coogan et al., 2016; Becker, 2020). Moreover, psychological inflexibility was found to mediate the relationships between both autistic traits and ADHD symptoms and HRQoL, depression, and pain interference. Psychological *flexibility* has been conceptualized as demanding “at least adequate performance” within the domain of executive function (Kashdan and Rottenberg, 2010), including cognitive processes with direct implications for e.g., goal oriented behavior, attentional control, decision making, and coping with new situations (Craig et al., 2016). Impairments in executive function constitute central deficits in ADHD and ASD (Craig et al., 2016). It is thus possible that the mediating role of psychological inflexibility in the current sample comes from its relation to actual behavioral performance (ability for adaptive functioning in the presence of pain or lack thereof) as an indirect measure of executive function. As previously mentioned, psychological *flexibility* may already constitute a resilience factor in pediatric chronic pain and these results indicate that psychological flexibility may be a resilience factor of greater importance to children with chronic pain and co-occurring ADHD symptoms or autistic traits. This notion is supported by research on pediatric chronic pain patients where functioning improved from pre- to post-treatment with Acceptance and Commitment Therapy (that specifically targets psychological flexibility) in patients both high and low in autistic traits and ADHD symptoms, and more so for patients higher in autistic traits (Balter et al., 2021).

A number of limitations should be considered when interpreting the findings of this study. First, the use of self-assessments of children only for functioning might have increased the risk of common method bias and the likelihood of finding significant relationships among included variables. The inclusion of objective measures of functioning, e.g., school absence, could thus have increased the validity of the findings. However, self-reported HRQoL has been found to be a valid predictor of recovery and quality of life over time for both healthy and non-healthy school-aged children (Riley, 2004). Second, information on medication status and regime of the participating children was not collected. Adjusting for such information could have further increased the validity of the findings. Third, the cross-sectional design does not allow for any causal or temporal inferences; and accordingly, fourth, the use of mediation analyses in longitudinal research designs, which was not the case in the current study that used a cross sectional design, allows for more stringent analysis of mechanisms of action (Cole and Maxwell, 2003). Therefore, any interpretations of possible causality with regard to the mediation results in the current study should be regarded as merely tentative.

Because of the significantly higher levels of depressive symptoms and pain interference, and significantly lower HRQoL, in participants with clinically significant autistic traits or symptoms of ADHD, clinical implications include the utility of routine screening of neurodevelopmental disorders in children and adolescents with chronic pain. Also, the mediating roles of insomnia and psychological inflexibility, although tentatively, suggest that these may be potential treatment targets for children with chronic pain and clinically significant traits and symptoms of ADHD and/or autism to improve functioning and HRQoL. Notably, few studies have yet specifically targeted insomnia in pediatric populations with comorbid physical health conditions such as chronic pain (Palermo et al., 2017), despite the documented increased risks for depression and functional disability from insomnia in pediatric chronic pain populations (Valrie et al., 2013).

The results from the present study warrant further research, to e.g., validate these findings in larger studies, evaluate the utility of tailored interventions, and examine a possibly shared pathophysiology of chronic pain and neurodevelopmental disorders, including dopamine dysfunction and sensory abnormalities.

## Conclusion

Although a number of previous studies have demonstrated a reduced level of functioning and HRQoL in pediatric chronic pain, this is the first study showing that autistic traits and ADHD symptoms significantly predict greater pain interference and depression, and reduced HRQoL in children with chronic pain. Children and adolescents with clinically significant traits of autism or ADHD symptoms further presented with significantly higher levels of depressive symptoms and pain interference, and lower HRQoL, than did the other children in the sample. The current results suggest psychological flexibility and good sleep as potential protective factors against ADHD/autism-related functional impairments in pediatric chronic pain, a hypothesis that could be further assessed in future research. These results underline the utility of screening for neurodevelopmental disorders in children with chronic pain, and may indicate insomnia, and skills related to psychological flexibility, such as attentional control and coping with demanding situations, as potential treatment targets to improve functioning and HRQoL in children with chronic pain and co-occurring symptoms of neurodevelopmental disorders.

## Data Availability Statement

The dataset of this article is accessible on request from the corresponding author.

## Ethics Statement

The studies involving human participants were reviewed and approved by the regional Ethical Review Board in Stockholm (Dnr: 2013/231-31-4). Written informed consent to participate in this study was provided by the participants’ legal guardian/next of kin.

## Author Contributions

CWL, TH, SB, ML, LH, and RW: conceived the study. CWL: data collection. CWL and RW: statistical analysis. CWL, LB, TH, ML, SB, LH, and RW: writing. All authors reviewed the manuscript.

## Conflict of Interest

SB has in the last 3 years acted as author, consultant, or lecturer for Medicine and Roche. He receives royalties for text books and diagnostic/intervention tools from Hogrefe publishers. TH receives royalties for text books from Hogrefe. RW receives royalties from Natur & Kultur. The remaining authors declare that the research was conducted in the absence of any commercial or financial relationships that could be construed as a potential conflict of interest.

## Publisher’s Note

All claims expressed in this article are solely those of the authors and do not necessarily represent those of their affiliated organizations, or those of the publisher, the editors and the reviewers. Any product that may be evaluated in this article, or claim that may be made by its manufacturer, is not guaranteed or endorsed by the publisher.
